# Estimating Dynamic Cellular Morphological Properties via the Combination of the RTCA System and a Hough-Transform-Based Algorithm

**DOI:** 10.3390/cells8101287

**Published:** 2019-10-21

**Authors:** Lejun Zhang, Yang Ye, Rana Dhar, Jinsong Deng, Huifang Tang

**Affiliations:** 1 Department of Pharmacology, School of Basic Medical Sciences, Zhejiang University, Hangzhou, Zhejiang 310058, China; 21718604@zju.edu.cn (L.Z.); 11818479@zju.edu.cn (R.D.); 2 College of Environmental and Resource Sciences, Zhejiang University, Hangzhou, Zhejiang 310058, China; yangye1993@zju.edu.cn

**Keywords:** xCELLigence real-time cell analysis, cell morphology, TGF-β, epithelial–mesenchymal transition, Hough transform

## Abstract

The xCELLigence real-time cell analysis (RTCA) system has the potential to detect cellular proliferation, migration, cytotoxicity, adherence, and remodeling. Although the RTCA system is widely recognized as a noninvasive and efficient tool for real-time monitoring of cellular fate, it cannot describe detailed cell morphological parameters, such as length and intensity. Transforming growth factor beta(TGF-β) induced the epithelial–mesenchymal transition (EMT), which produces significant changes in cellular morphology, so we used TGF-β to treat A549 epithelial cells in this study. We compared it with lipopolysaccharide (LPS) and cigarette smoke extract (CSE) as stimulators. We developed an efficient algorithm to quantify the morphological cell changes. This algorithm is comprised of three major parts: image preprocessing, Hough transform (HT), and post-processing. We used the RTCA system to record the A549 cell index. Western blot was used to confirm the EMT. The RTCA system showed that different stimulators produce different cell index curves. The algorithm determined the lengths of the detected lines of cells, and the results were similar to the RTCA system in the TGF-β group. The Western blot results show that TGF-β changed the EMT markers, but the other stimulator remained unchanged. Optics-based computer vision techniques can supply the requisite information for the RTCA system based on good correspondence between the results.

## 1. Introduction

Morphological properties are used to distinguish different kinds of cells or the same cell in different stages to help with understanding molecular cell fate [1]. Many studies have demonstrated that treatment with transforming growth factor beta (TGF-β) results in a significant change in cellular morphology; epithelial cells are changed from a typical cobblestone type to slender fibroblast-like mesenchymal cells [2,3]. This process is also called epithelial–mesenchymal transition (EMT). EMT was first described by Hay [4] and plays an important role in the processes of chronic inflammation, tissue reconstruction, tumor migration, and fibrosis diseases [5,6]. Thus, morphological changes are an imperative optical indicator to illustrate the effect of TGF-β in the EMT process.

Numerous studies have described changes in cell shape by direct microscopic observations [7,8]. Some reports have observed morphological changes of cells using immunofluorescence assay [2,9]. A new real-time apparatus called the xCELLigence real-time cell analysis (RTCA) system has become popular for detecting the proliferation, migration, cytotoxicity, adherence, and remodeling of cells, and drug screening [10,11,12]. It is widely recognized as a noninvasive and efficient tool for real-time monitoring of cellular fate [13,14]. The system mainly detects impedance change using an E-plate 96, which is covered with a metal electrode for signal induction. When cells attach to or detach from the surface electrodes of the E-plate 96, electronic impedance occurs [15]. For example, changes in shape, proliferation, and adhesion rate of cells produce different impedances over time [16,17]; the impedance signal can be transformed into a cell index, presented as colorful curves that provide an approach to visualize the biological status of cells [15]. However, it cannot detail the morphological parameters of cells, such as length and intensity. Therefore, developing a novel method to quantify the morphological cell change using optics-based computer vision techniques is necessary. 

Computer vision techniques have gained popularity for detecting the characteristics of cells, including single-cell shape, cell number, and categories using fluorescent dyes [18,19]. Few reports, however, have proposed methods to extract the morphological parameters of TGF-β-stimulated A549 cells using an algorithm. Thus, we designed an algorithm to acquire the parameters of cells according to the images captured by an inverted microscope. The development of the algorithm is based on MATLAB R2014a (MathWorks, Natick, MA, USA), which is a programming platform that can be used for image processing. Our framework is divided into three major parts: image preprocessing, Hough transform (HT), and post-processing. HT is the core step, providing an effective method to detect straight and curved lines in an image [20]. The sound mathematical theory of HT has been applied in many disciplines, such as the detection of lane lines [21], the calculation of the height of trees [22], and red blood cell counts [18,19]. In this study, we used the HT method to extract and mark the boundaries of cells. To develop the best procedure, we applied some modifications after the HT process to quantify the cells’ number and length. 

Based on the above considerations, in this research, we first found that the EMT process activation is induced by TGF-β at different time points and further confirmed the expression of EMT markers by Western blot. Then, we applied the RTCA system to understand the relationship between the morphological properties and the typical cell index. An effective MATLAB-based algorithm was developed to extract and quantify the cells’ structural parameters from the cell images. After a comparison of the two methods, the morphological characteristics obtained by the algorithm provide a good supplement to the requisite information of the RTCA system.

## 2. Materials and Methods

### 2.1. Reagents

TGF-β was purchased from PeproTech (PeproTech Inc., Rocky Hill, NJ, USA). Lipopolysaccharide (LPS) was purchased from Sigma-Aldrich. (St. Louis, MO, USA). Research-grade (3R4F) Marlboro cigarettes containing 9.5 mg tar and 0.73 mg nicotine per cigarette was used in this study. Cigarette smoke extract (CSE) was prepared according to the methods reported in previous studies [23,24]. This medium was considered as “100% CSE”, and then diluted to different concentrations with fresh culture medium for experiment.

### 2.2. Cell Culture

The A549 human NSCLC cell line was purchased from ATCC (CCL-185, Rockville, MD, USA). For conventional passage, cells were cultured in RPMI 1640 medium, supplemented with 2 mM l-glutamine (Corning Life Science, Manassas, VA, USA), 10 mM HEPES, 100 U/mL penicillin, 100 U/mL streptomycin, and 10% (*v/v*) fetal bovine serum (FBS) (Sigma-Aldrich, St. Louis, MO, USA) and incubated in 5% CO_2_ at 37 °C for 24 h. For cell starvation, cells were cultured in RPMI 1640 medium containing 0.5% (*v/v*) FBS for 24 h. After cell starvation, the medium was changed, cells were cultured in the same low serum containing RPMI 1640 medium for drug administration during the next 48 h: TGF-β (10 ng/mL), LPS (100 ng/mL and 500 ng/mL), and CSE (1% and 2%). The cells without stimulation were used as the control. During the stimulation, we captured pictures under an optical microscope (#OlympusCKX31, Olympus Corporation, Tokyo, Japan) and then harvested cells for Western blot analysis. 

### 2.3. Cell Index Assay in xCELLigence RTCA Single-Plate (SP) System

The xCELLigence RTCA SP system (Roche Applied Science, Basel, Switzerland) consists of three parts: (1) an RTCA analyzer (ACEA Biosciences Inc., San Diego, CA, USA, model W380, catalog number: 00380601510) used to measure, process, and analyze the impedance (cell index), which was detected by sensor electrodes in E-plate 96 (ACEA Biosciences, catalog number: 05232376001); (2) the RTCA SP Station (ACEA Biosciences, catalog number: 05229057001) was a plate holder that was placed inside of the incubator and connected to the RTCA analyzer; and (3) an RTCA control unit (ACEA Biosciences, catalog number: 05454417001) was preinstalled with RTCA software (ACEA Biosciences, version numbers 1.2.1.1002 and 2.1.0) to evaluate the data. To measure the background, we placed 50 µL RPMI 1640 medium in the E-plate 96 and placed it in the incubator at 37 °C, 5% CO_2_. Then, we added 100 µL A549 cell suspension (3000 cells/well) with 10% FBS RPMI 1640 medium and maintained the plate at room temperature (RT) for 30 min to confirm the suspension in the bottom part of the plate. After 24 h incubation, the medium was changed with 100 µL of 0.5% FBS RPMI 1640 for cell starvation, and the cell index was recorded every 5 min for 24 h. Then, the cells were stimulated with TGF-β (10 ng/mL), LPS (100 ng/mL and 500 ng/mL), and CSE (1% and 2%) and monitored every 5 min to obtain the cell index for 48 h. Every independent experiment was performed in triplicate. The interval slope was calculated automatically by the RTCA software to evaluate the rate of cell index change. To demonstrate the effect of treatments, the cell index was normalized to an equal value at the normalization time point. 

### 2.4. Western Blot Analysis

Proteins were extracted by radio immunoprecipitation assay (RIPA) lysis buffer (including protease inhibitor cocktail, Phenylmethanesulfonyl Fluoride (PMSF), and phosphatase inhibitor) and centrifuged at 13,000× *g* at 4 °C for 15 min. Protein concentrations were determined using Bio-Rad reagent (Bio-Rad Inc., Hercules, CA, USA). The 5× loading buffer (Beyotime Inc., Shanghai, China) was added to the proteins and boiled at 120 °C for 5 min. Dodecyl sulfate sodium salt (SDS)-Polyacrylamide gel electrophoresis (PAGE) (SDS-PAGE) gels were prepared at 8%, 10%, or 12%. Thirty micrograms of proteins were electrophoresed (30 V for 30 min, 70 V for 40 min, and 130 V for 30 min) and then transferred to nitrocellulose (NC) membranes (300 mA for 90 min). After, the membranes were blocked with blocking buffer (5% bovine serum albumin (BSA)) for 1 h and incubated with following primary antibodies at 4 °C overnight. Rabbit anti-GAPDH (# db106, 1:50000) and rabbit alpha smooth muscle actin (α-SMA) (#db2140, 1:8000) were purchased from Digbio (Hangzhou, China). Rabbit anti-fibronectin (#GTX112794, 1:1000) and rabbit anti-E-cadherin (#GTX100443, 1:1000) were purchased from Gentex (San Antonio, TX, USA). After, the membranes were washed with 1× Tris-buffered saline and Tween-20 (TBST) three times, and then incubated with the secondary antibody (1:5000) (IRDye 800CW goat anti-rabbit; IRDye 680CW goat anti-mouse (LI-COR Biosciences, Cambridge, U.K.) for 1.5 h at room temperature. The membranes were washed with 1× TBST three times and then imaged with Odyssey CLx infrared imaging system (LI-COR Biosciences, Cambridge, U.K.). The bands were quantified using Imagine Studio Version 5.2 software (LI-COR Biosciences, Cambridge, U.K.), and GAPDH was used to normalize the target of the proteins.

### 2.5. Detection of Morphological Parameters

**Figure 1:**
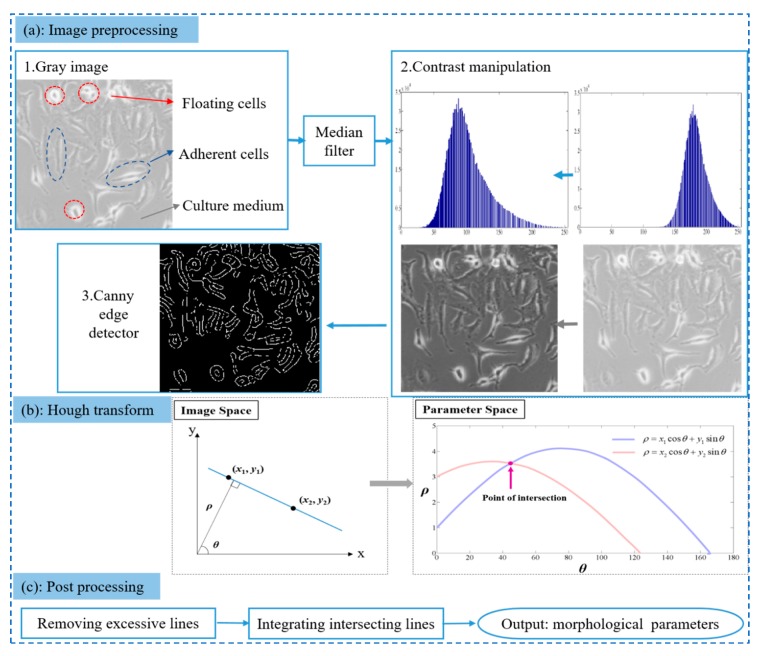
The framework of the digital image process. (**a**) Image preprocessing includes gray transformation, median filter, contrast manipulation, and canny edge detection; (**b**) Hough transform (HT) demonstrates the transformation between image space and parameter space; and (**c**) post-processing includes removing excessive lines and integrating intersecting lines.

The images of the cells recorded by a digital camera are fairly complicated due to the different cell growth stages, cell numbers, and the mixtures of various substance (adherent cells and floating cells) (Figure 1). All of that added the difficulty of image processing. To ensure the robustness of our method in complex situations, we designed the following three processes: (a) image preprocessing, (b) HT, and (c) post-processing (Figure 1). These processes emphasize the important information in the image and ignore the other noise. Our framework was realized by programming based on MATLAB R2014a.

#### 2.5.1. Image Preprocessing

The original three-channel color images (red, blue, green (RGB)) were first transformed into single-channel gray images. We then chose a median filter to remove the noise to preserve the sharp edge of cells while efficiently removing the salt-and-pepper noise [25]. The cells in the image are relatively darker since the light transmittance of the cytoplasm is lower than that of the culture medium. Therefore, the images should be further enhanced with contrast manipulation by highlighting the shape of cells; a cube function was also implemented to stretch the gray range as follows:

*F*(*i,j*) = [*G*(*i,j*)]^3^, (1)

where *G*(*i,j*) represents the normalized value of the pixel in the denoised gray image and *F*(*i,j*) is the enhanced value of the pixel. The cube function has a slower slope where the value is small; thus, cells with a low gray level would be stretched after enhancement. As such, the difference between the cells and the culture medium becomes apparent, as do the signals of cell boundaries (Figure 1a). The final step in the preprocessing is characterizing cells’ boundaries based on a canny edge detector [26], which is a popular non-parametric edge detection approach with three features: good detection, good localization, and low spurious response. This method finds edges by looking for the local maxima of the image gradient, and the gradient is calculated by the derivative of a Gaussian filter. The edge image (Figure 1a) contains the main morphological information of cells and removes other useless signals, including the cytoplasm and culture medium.

#### 2.5.2. Hough Transform (HT)

The edge images obtained from preprocessing are binary images, including boundaries of cells (white) and background (black). The boundaries of cells are relatively randomly distributed due to cells’ disordered locations. Therefore, we applied HT to locate and quantify every cell boundary in the edge images. HT is an effective method to detect straight and curved lines. A straight line in x,y image space can be represented as angle–radius (*θ–ρ*) in parameter space [20]:

(2) ρ=x⋅cosθ+ysinθ

As shown in Figure 1b, parameter *ρ* represents the algebraic distance between the line and the origin, and *θ* is the angle between the normal line and the *x*-axis. If two points {(*x*_1_, *y*_1_), (*x*_2_, *y*_2_)} are collinear, they have the same parameters (*θ*–*ρ*). After transforming the two points into the sinusoidal curves in the *θ–ρ* plane, the curves with an intersection point correspond to collinear points in *x,y* space (Figure 1b). To specify the unique relationship between the line and the parameters, *θ* is restricted to the interval [0,π). As such, all the points in the *x,y* image space can be converted into curves in parameter space, and curves concurrent at (*θ_i_–ρ_j_*) would be voted in accumulate array [H_ij_]. [H_ij_] was used to record the number of points in the image space that intersected at the same parameters (*θ_i_–ρ_j_*). Finally, we were able to determine all the straight lines in the image space according to the [H_ij_] information. In our case, only lines longer than 20 pixels are marked and recorded in an image because a lower threshold can easily be affected by image noise. HT is also able to avoid the obstruction introduced by floating cells in the image. To be specific, floating cells in the image are bright and rounded (Figure 1a), distinguishing them from other cells. However, the aim of HT is to detect a linear pattern, so rounded floating cells would be neglected.

#### 2.5.3. Post-Processing

**Figure 2:**
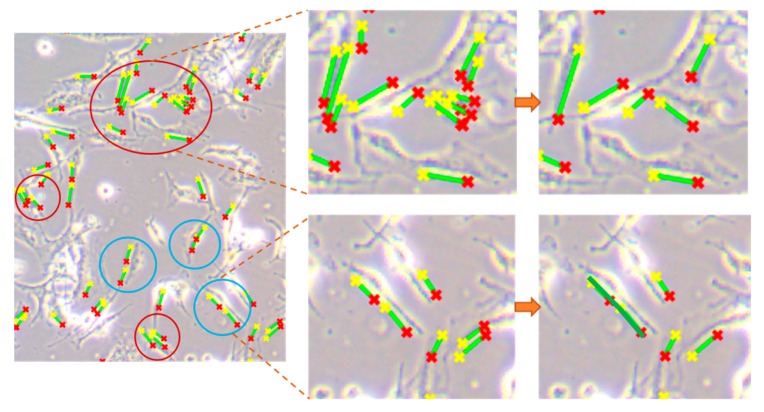
Two kinds of detection before and after post-processing. The red circles represent excessive lines for marking cells, and the blue circles represent lines with points of intersection.

After all the straight lines in the images are marked, some unreasonable results remain when quantifying the morphological characteristics of the cells. Hence, we divided the unreasonable detections into two categories: (1) one cell might be marked by approximate parallel lines more than once, as shown in Figure 2 (red circles); (2) a slender cell might be described by several discrete lines (blue circles), where the lines should be joined into a longer line to represent the real attributes of the cell. Since these issues can impact the accuracy of the results, we had to correct the detections after Hough transformation. First, if the minimal distance between two lines was fewer than 15 pixels and with an included angle less than 20°, the shorter line would be removed, while retaining the longer one to mark the cell. Secondly, lines with intersection points or very close were integrated as a longer piecewise line to represent a cell. Correcting the above unreasonable detections can guarantee the accuracy of cell quantification. Finally, the line lengths of cells and the number of lines were output as morphological parameters to indicate the cells’ dynamic changes under different treatments. We selected several typical images to validate the results from the algorithm and manual detections (Figures S1 and S2), provided the scatter plots of real length and detected length (Figure S3) and compared the error of detected length among different groups (Figure S4).

### 2.6. Statistical Analysis

The cell index was used for real-time cell fate assessment; the slope of the cell index and normalized cell index were calculated automatically by the RTCA software package 2.1.0(ACEA, Biosciences Inc., San Diego, CA, USA). The numerical data are reported as the mean ± SD. The Western blot analysis data are reported as the mean ± standard error of the mean (SEM). The morphological parameters were obtained based on typical images with numerical form, and the data are shown as the mean ± SD. Statistical differences between the different groups were evaluated using one-way ANOVA with Bonferroni’s multiple comparison test with GraphPrism5 software (GraphPad Software Inc., San Diego, CA, USA); *p* < 0.05 was considered significant. 

## 3. Results

### 3.1. Phenotypic Changes in the TGF-β-Induced EMT Process in A549 Cells

**Figure 3:**
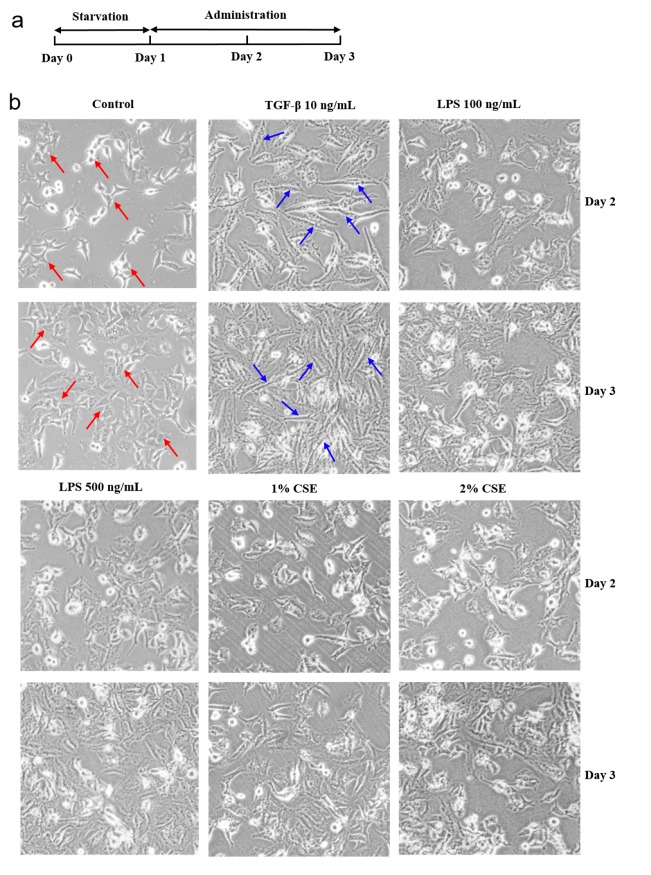

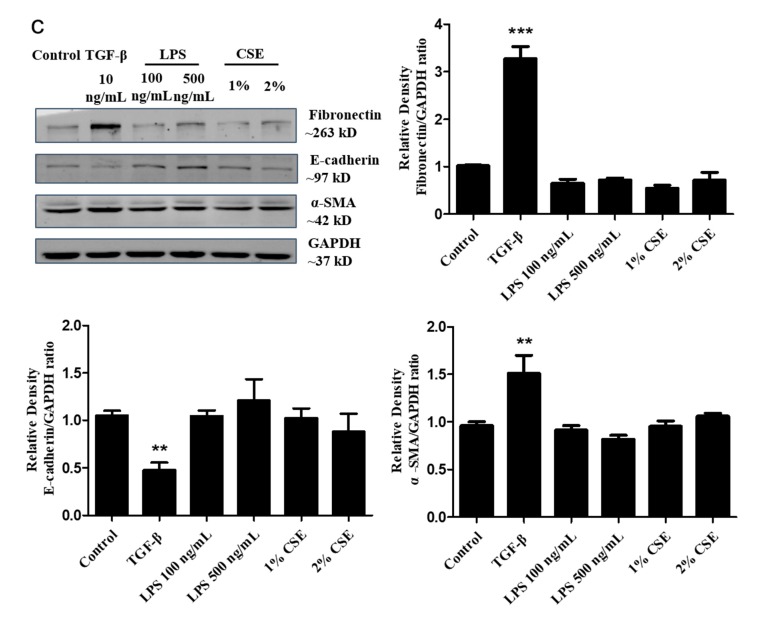
Changes in morphology and biomarkers in the transforming growth factor beta (TGF-β)-induced epithelial–mesenchymal transition (EMT) process of A549 cells. (**a**) Schematic treatment points of experimental design. After serum starvation for 24 h, cells were treated with TGF-β (10 ng/mL), lipopolysaccharide (LPS) (100 and 500 ng/mL), and cigarette smoke extract (CSE) (1% and 2%) for 48 h. (**b**) Representative images (original magnification 200×). Red arrows indicate a part of the typical cell shape (cobblestone type) in the control group, and blue arrows indicate a part of the fibroblast type of A549 cells after stimulation by TGF-β. (**c**) Representative bands and quantitative analysis of EMT markers (fibronectin, E-cadherin, and alpha smooth muscle actin (α-SMA). The expressions were detected by Western blot. GAPDH was used as the loading control. *** *p* < 0.001, ** *p* < 0.01 vs. the control group, *n* = 3.

To investigate the morphological changes of A549 cells, we stimulated A549 cells with TGF-β (10 ng/mL), LPS (100 ng/mL; 500 ng/mL), and CSE (1% and 2%). Cells were treated following the timeline in Figure 3a. We found that after the induction of TGF-β, A549 cells changed to the fibroblast type, but this change was not observed in the LPS and CSE groups (Figure 3b). These results suggest that TGF-β can induce distinct morphological changes in A549 cells. Some studies revealed that TGF-β initiates the EMT process by altering the expression of genes, including E-cadherin [27], vimentin [28], and α-SMA [29], which leads to a loss of cell–cell junctions [7], cytoskeletal reconstruction [30], and an increase in cell invasion [31]. To further confirm the biological changes of A549 cells induced by TGF-β, LPS, and CSE, we detected the EMT markers using Western blot, which showed that TGF-β significantly upregulated the expression of fibronectin and α-SMA but downregulated E-cadherin compared with the control group (*p* < 0.01; Figure 3c). However, no significant differences were observed in the LPS and CSE groups compared with the control group. Taken together, the TGF-β-induced EMT process is responsible for the distinct morphological changes in A549 cells.

### 3.2. Real-Time Detection of Cell Index in A549 by xCELLigence RTCA SP System

**Figure 4:**
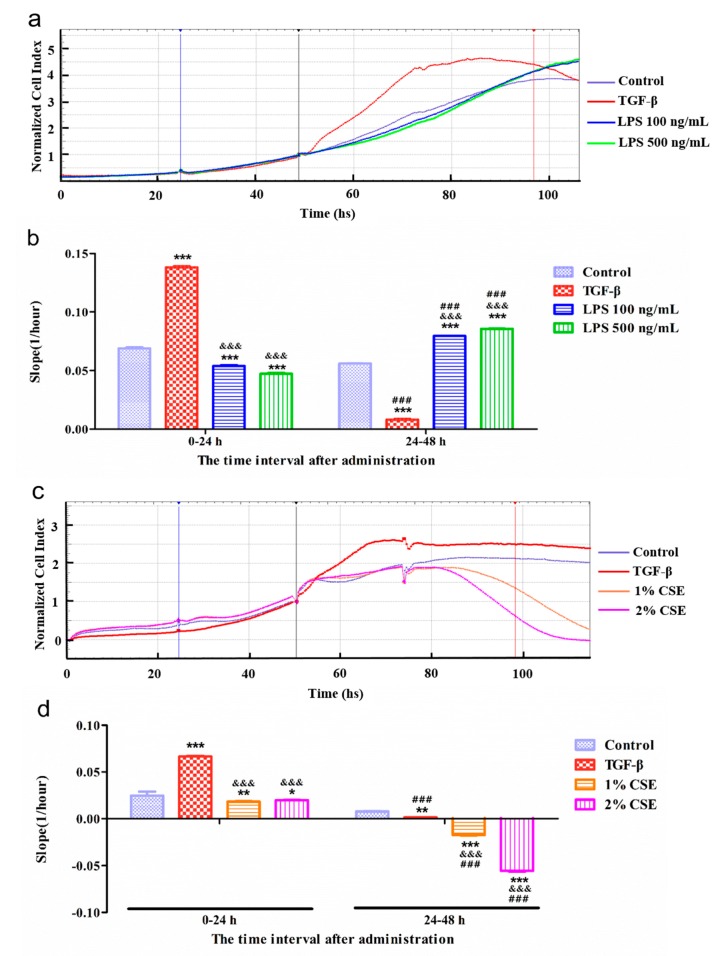
Real-time detection cell index of A549 with different treatments in the xCELLigence real-time cell analysis (RTCA) single-plate (SP) (RTCA SP) system. (**a**) A549 cells were treated with TGF-β and LPS (100 and 500 ng/mL). The serum starvation step for 24 h starts from the blue vertical line to the black vertical line in the timeline. The time interval between the black and red vertical lines represents administration for 48 h, and the black vertical line is the time to normalize the cell index. Representative curves of the normalized cell index. (**b**) The representative interval slope of the TGF-β and LPS groups. (**c**) A549 cells were treated with TGF-β and CSE (1% and 2%). The conditions were the same as above. (**d**) The representative interval slope of the TGF-β and CSE groups. For the LPS experiment, *n* = 9; for the CSE experiment, *n* = 6. *** *p* < 0.001, ** *p* < 0.01, * *p* < 0.05 vs. control group. ### *p* < 0.01 24–48 h groups vs. 0–24 h groups. &&& *p* < 0.001 vs. TGF-β group in different time intervals.

To investigate the changes in the cell index, we stimulated A549 cells with TGF-β (10 ng/mL), LPS (100 and 500 ng/mL), and CSE (1% and 2%), in the xCELLigence RTCA SP system. As shown in Figure 4a,c, the normalized cell index curve of the TGF-β group showed an arch structure and stayed at the peak position at 24–48 h, whereas the LPS groups had a curve similar to the control group. After administration 24 h, the slope of the TGF-β group significantly increased, whereas those of the LPS groups decreased compared with the control group (Figure 4b). However, in the next 24 h, the tendency of the curves changed when compared to the control group. The TGF-β group was significantly decreased, whereas the LPS groups were still increasing. Additionally, we found that the cell index in the CSE groups decreased in a dose-dependent manner (Figure 4c,d). For we observed the shape was the arch structure in TGF-β group, we further confirmed the shape by statistical analysis as showed in Figures S5–S7. The variations in the curve were significantly different in Figure S7.

Altogether, these results suggest that the EMT process stimulated by TGF-β has a specific tendency in terms of the cell index curve, providing a novel approach to recognize the different stimulations in vitro by using the xCELLigence RTCA SP system. 

### 3.3. Morphological Changes of A549 Cells Detected by Image Processing Techniques

**Figure 5:**
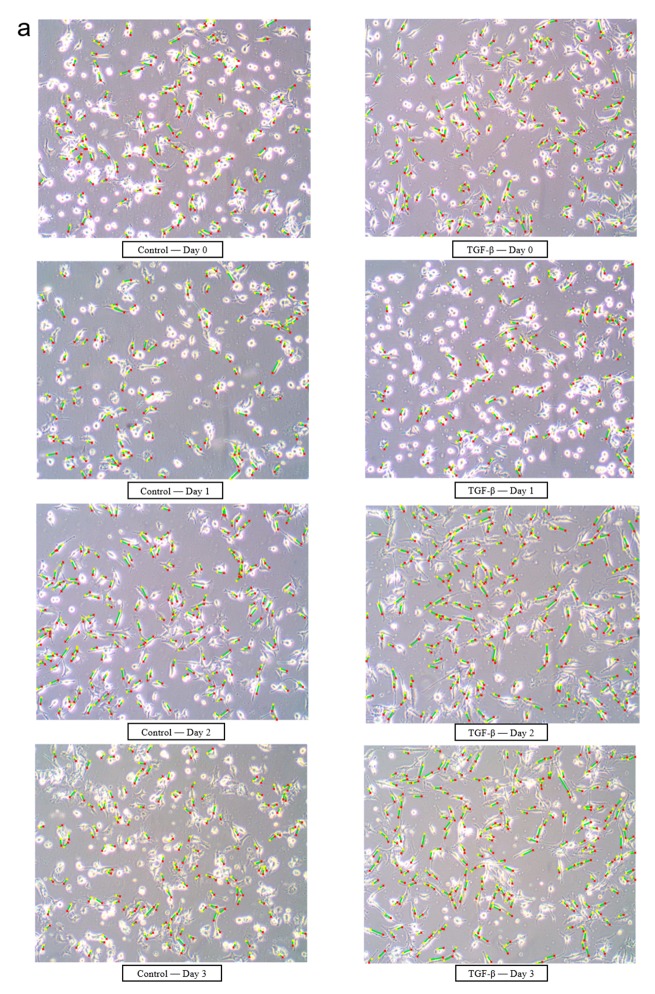

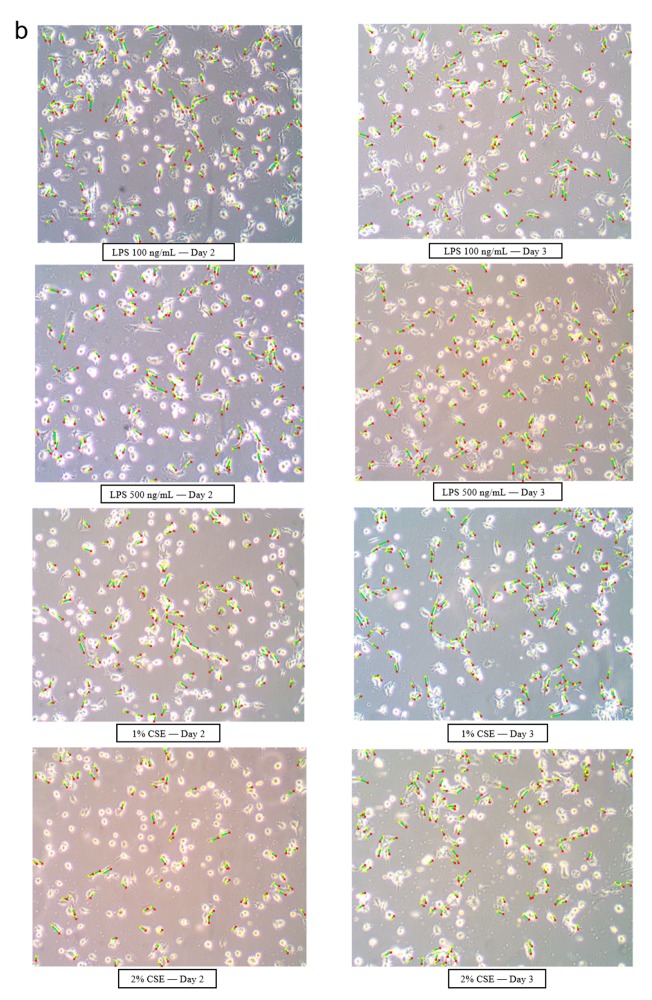
Morphological analysis of A549 cells using image processing techniques. The original pictures were obtained by optical microscope (200× magnification). (**a**) Representative images of A549 cells in the control and TGF-β groups from days 0 to 3. (**b**) Representative images of A549 cells in LPS (100 and 500 ng/mL) and CSE (1% and 2%) groups on days 2 and 3 (green: cell length; red: start point; yellow: end point).

To further understand the cell morphology, we applied image processing to extract the cells’ parameters via the following different treatments in a time-dependent manner. As shown in Figure 5a, the initial images (Control—Day 0 group and TGF-β—Day 0 group) and 24 h images (Control—Day 1 group and TGF-β—Day 1 group) in both the control group and the TGF-β group were relatively similar due to the cells in TGF-β group having had no treatment before 48 h. On day 1, the numbers of cells were reduced in control and TGF-β groups after serum starvation. On day 2, the shape of the cells in the TGF-β group started to narrow, a significant difference compared with the images of the control group. However, the LPS (100 and 500 ng/mL) and CSE (1% and 2%) groups did not show a similar morphology to TGF-β on days 2 or 3 (Figure 5b).

### 3.4. Quantification of Cells’ Parameters using Image Processing Techniques

**Figure 6:**
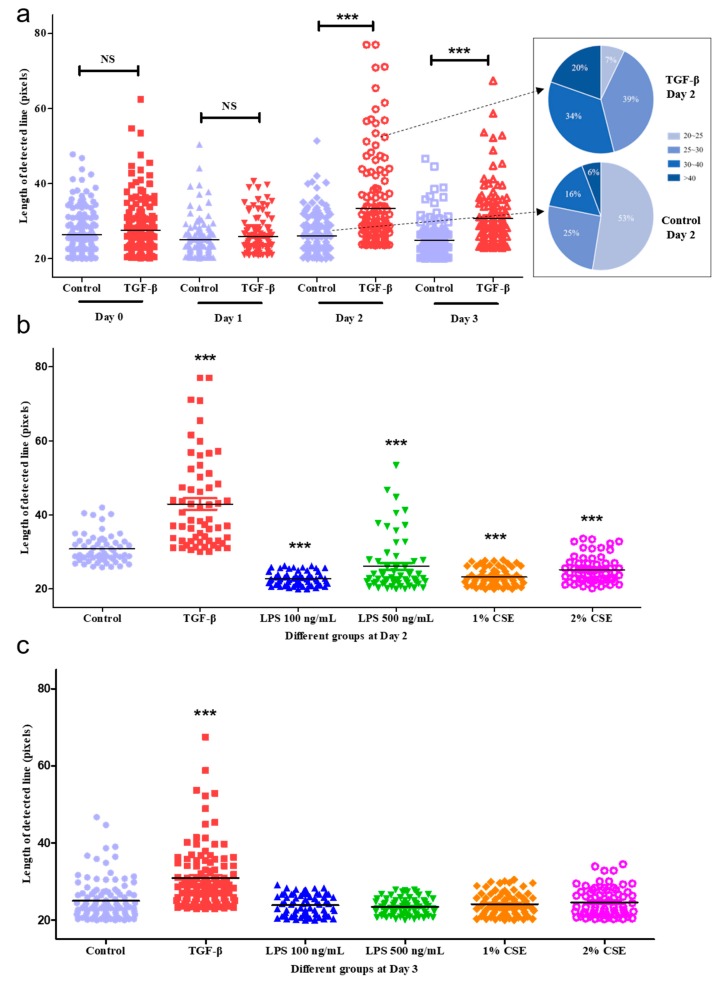

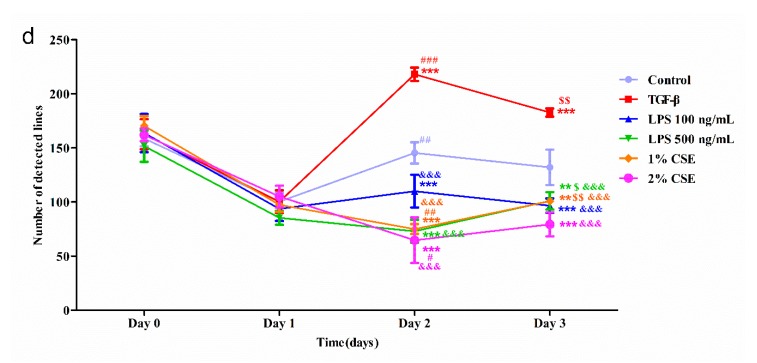
The quantification of the length and quantity of detected lines. (**a**) A scatter dot plot of the length of the detected lines between the control and TGF-β groups. Pie charts represent the percentages of different length levels on day 2: 20–25, 25–30, 30–40, and >40 pixels. (**b**) A scatter dot plot comparing the six different groups (control; TGF-β; LPS 100 ng/mL and 500 ng/mL; 1% and 2% CSE groups) on day 2. (**c**) A scatter dot comparing the six different treatments on day 3. (**d**) A line graph focused on the number of lines detected from day 0 to 3 in the six groups. *n* = 3. *** *p* < 0.001, ** *p* < 0.01 vs. control group in different time points. ### *p* < 0.001, ## *p* < 0.01, # *p* < 0.05 Day 1 vs. Day 2. $$ *p* < 0.01, $ *p* < 0.05 Day 2 vs. Day 3. &&& *p* < 0.001 vs. TGF-β group in different time points.

We calculated all the lengths of the detected lines in every group in the time series and plotted them as scatters in Figure 6a. The scatters show many more lines, distributed at a range of high length values in the TGF-β group on days 2 and 3. In detail, the percentage of lines with more than 40 pixels in the TGF-β—Day 2 group was 20%, which was higher than that in the Control—Day 2 group (6%). Fifty-three percent of the lines in the Control—Day 2 group had fairly small lengths (20–25); these were also smaller than in the TGF-β—Day 2 group. The above results indicated that the shape of cells significantly changed with or without treatment, which confirmed our expectation that the long and narrow cells in TGF-β had an obvious linear characteristic. We also compared the detected line lengths among the different treatments on days 2 and 3 (Figure 6b,c). This suggested that the lengths of the three groups (LPS 100 ng/mL, 1% CSE, and 2% CSE) were shorter than those of the control group on day 2, whereas the TGF-β group’s lengths were the longest among all the groups. The above results had a similar tendency as the cell index obtained from the RTCA SP system (Figure 4). The number of lines detected represented the number of cells, which is a vital parameter for identifying the proliferation of cells, as shown in Figure 6d. Our results illustrated that, after the stimulation with TGF-β, the cell number increased sharply compared to the control group until day 2, and in the next 24 h, the number gradually decreased. In conclusion, the TGF-β-induced EMT process in A549 cells could be quantified as a conspicuous change in cell length and cell quantity using image processing techniques, which supplemented the results of the RTCA system and helped us to understand the details of the cell index curve.

## 4. Discussion

The RTCA system is not only a well-recognized modern technique but also a novel, noninvasive, and efficient tool to dynamically record the biological status of cells [32]. Morphological variation is one of the vital parameters that contributes to the change in the cell index and provides a rational approach to evaluate the EMT process using the change in the cell index. Staršíchová et al. demonstrated that the RTCA system as a real-time apparatus provides dynamic monitoring of cellular fate induced by TGF-β [10]. In this study, we found that a high slope, or the change to an arch structure after the stimulation with TGF-β (10 ng/mL) by the xCELLigence RTCA SP system, meant the cell index increased more rapidly than in other groups. Based on the principle of the xCELLigence RTCA system, cell morphological change or cell proliferation could induce a change in impedance, which results in a change in the cell index. For the TGF-β group, the high slope of the cell index may be related to the cell morphological change and cell proliferation. Computer vision techniques (image processing) were proposed to automatically quantify the exact morphological parameters, i.e., the length and number of cells. The results indicated that TGF-β-treated A549 cells became longer than the cells in the control group at 24 and 48 h after stimulation. The number of lines detected in Figure 6d also suggested that the cell proliferation of the TGF-β group increased rapidly on day 2. The results of the above two techniques have similar tendencies, so we think that the change in cells’ length and number contributes to the unique arch structure. These changes are related to the intracellular EMT process in A549 cells after the induction w TGF-β as detected by Western blot, shown as downregulation of epithelial cell markers (E-cadherin) and upregulation of mesenchymal cell markers (fibronectin or α-SMA). Recent research has explained that the regulation of these EMT-related proteins is involved in several signaling pathways, such as drosophila mothers against decapentaplegic protein (Smad) [33], Protein kinase B (Akt)- mammalian target of rapamycin (mTOR) (Akt-mTOR) [34], and receptor tyrosine kinases (RKTs) [35].

Some evidence has also suggested that besides TGF-β, other important stimulators can induce EMT, such as LPS [36,37] and CSE [38,39], at high concentrations in cancer cells. In our study, LPS (100 and 500 ng/mL) and CSE (1% and 2%) did not affect the EMT process in A549 cells, whereas TGF-β had a positive effect. Compared with the high concentrations reported in the literature, our concentration is very low, so our data suggest that the lower concentrations of LPS and CSE might not induce the EMT process. The cell index curves detected by the xCELLigence RTCA system between the LPS and CSE groups were different, although they could not induce the EMT process. It was worth exploring the detailed cell changes using image processing techniques. Unlike the TGF-β group, after the treatments with LPS and CSE at different concentrations, as shown in Figure 6, the number of detected lines was not consistent with the results in the xCELLigence RTCA SP system. There were some uncontrolled parameters, such as: (1) it was difficult to detect the original numbers of cells when they overlapped with each other; and (2) the image processing was based on the cell images, which contained only some of the cells, whereas the xCELLigence RTCA SP system detected the impedance of the whole area in a well. Therefore, the results of image processing would be influenced by the choice of image view. 

Besides these parameters, we speculated that some other possible reasons were as follows: (1) Various parameters contribute to changes in the cell index, such as morphological changes, proliferation, and adhesion in the xCELLigence RTCA SP system. Thus, the cell number might not be the main factor controlling the cell index from the LPS or CSE groups. (2) The HT-based algorithm is more sensitive to morphological changes, i.e., the signals from the images of TGF-β-induced cells would be detected much more easily and accurately. The relationships between the above parameters still require further research.

In the framework of image processing, the HT can effectively locate the edge of cells under complex surroundings, which not only include the dynamic shape and number of cells but also various sources of noise, such as floating cells, non-uniform illumination, and blurry cell boundaries. However, the focus of HT is on linear patterns, so rounded floating cells would be neglected. More importantly, post-processing after HT is necessary for guaranteeing that one cell is reasonably marked by a single line or multiple lines. HT has a sound theoretical basis for detecting lines, so it is simple and effective to apply in computing processes.

In our study, we focused on the distinct morphological changes in the EMT process of A549 cells. The xCELLigence RTCA SP system can visualize the EMT process, and image processing techniques can be used quantify the length and number of cells. Based on our results, the xCELLigence RTCA SP system will provide a new approach for screening therapeutic drugs according to the changes in the cell index curves of the TGF-β-induced EMT process. Further study need improve the algorithm method, such as introducing dynamic curve functions for identifying various cell shapes.

## Supplementary Materials

The following are available online at https://www.mdpi.com/2073-4409/8/10/1287/s1. Figure S1: The visual interpretation of cells and algorithm detected cells in five cases. Figure S2: The number of detections including valid detections (Light blue color), wrong detections (Orange color) and missing detections (Gray color) in five groups. Figure S3: The scatter plots of real length and detected length in five groups. Figure S4: A scatter dot plot of length error comparing the five different groups (TGF-β; LPS 100 ng/mL and 500 ng/mL; 1% and 2% CSE groups) at day 2. Figure S5: The specific value of three points (the center of arch structure, the previous and next points) in two independent experiments (*n* = 3). Figure S6: The specific value of normalized cell index at Day 1 to Day 3 (*n* = 3). Figure S7: The slope at 0–24 h and 24–48 h after administration.
