# Supplementary material for: Estimating Dynamic Cellular Morphological Properties via the Combination of the RTCA System and a Hough-Transform-Based Algorithm
Source: Cells. 2019 Oct 21;8(10):1287. doi: 10.3390/cells8101287 (PMC6829879; doi:10.3390/cells8101287)
Supplement: Supplementary file 1 [file cells-08-01287-s001.pdf]

Supplementary Materials

Part 1: Validation of Method

In order to validate the results of detected cells by the proposed method, we selected five typical images (TGF-β, 1% CSE, 2% CSE, LPS 100 ng/mL and LPS 500 ng/mL) at Day 2 to check the cells one by one based on visual interpretation. In this way, almost 600 cells in five cases were checked and we calculated the total cell number, valid detected cell number, invalid detected number, true cell length, detected cell length and the error between them. Invalid detected number included wrong detections and repeated detections.

As shown in Figure S1, the real cells were marked as blue ones by manual plotting and then printed on the paper as well as the detected images. We checked each cell in the real images and its corresponding cell in the detected images to identify whether the cell had been detected. The wrong detections were also marked as black arrows.

a:1% CSE-Day 2

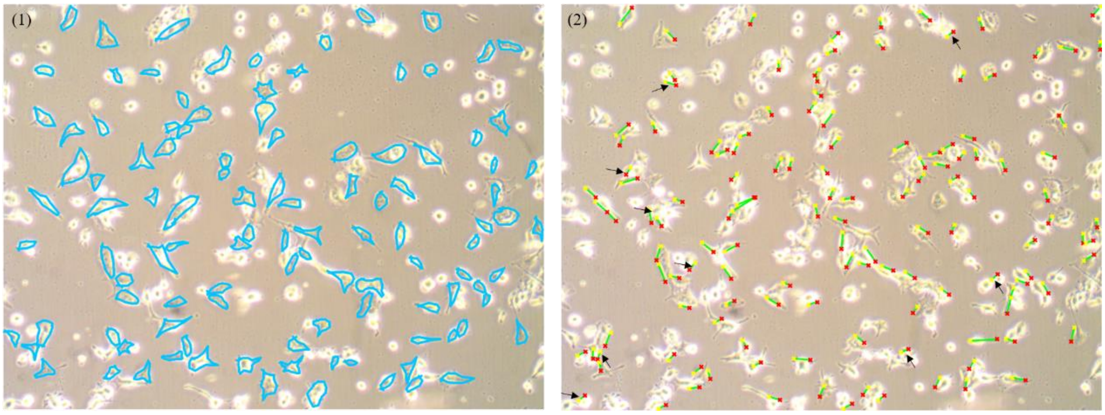

b:2% CSE-Day 2

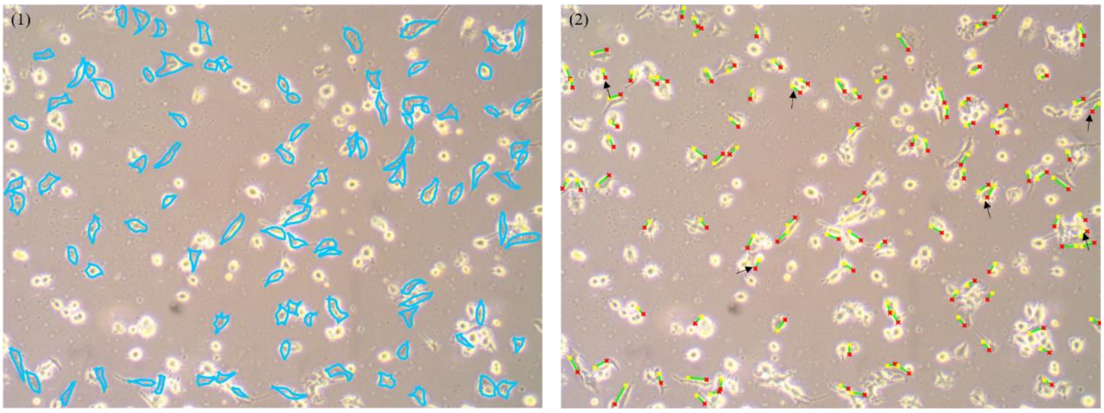

c:LPS 100 ng/mL-Day 2

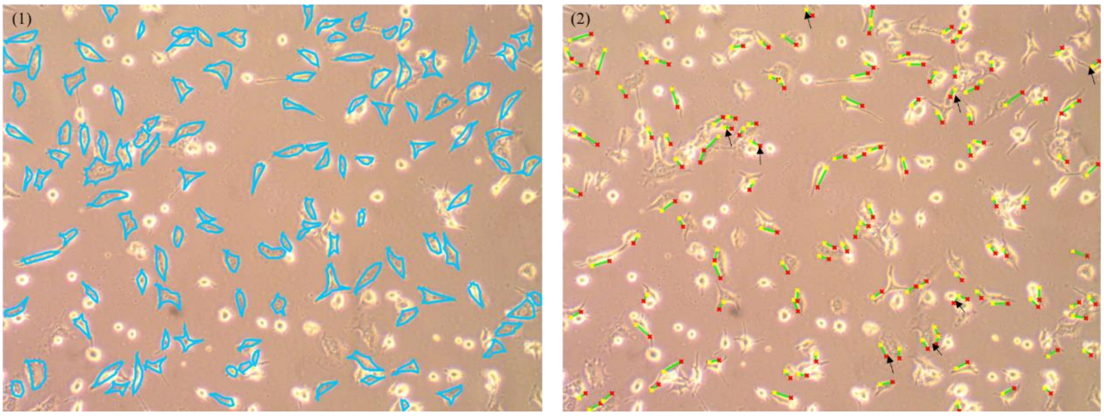

d:LPS 500 ng/mL-Day 2

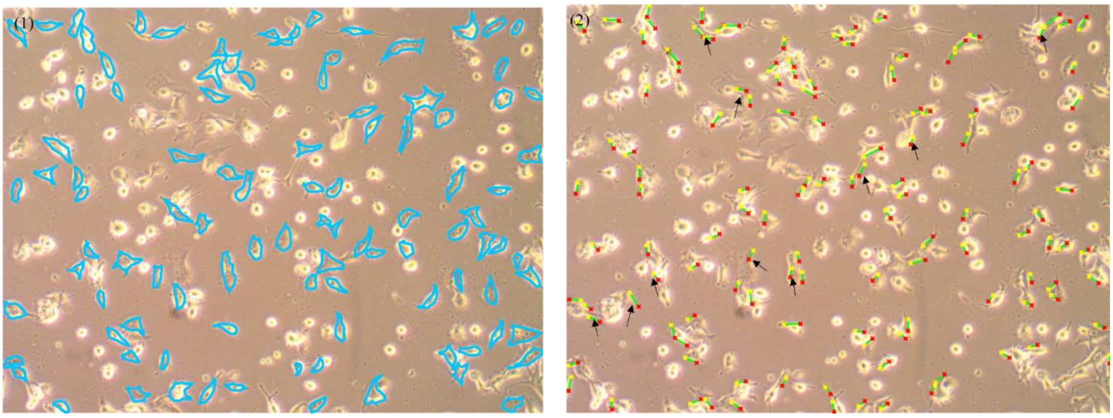

e:TGF-β-Day 2

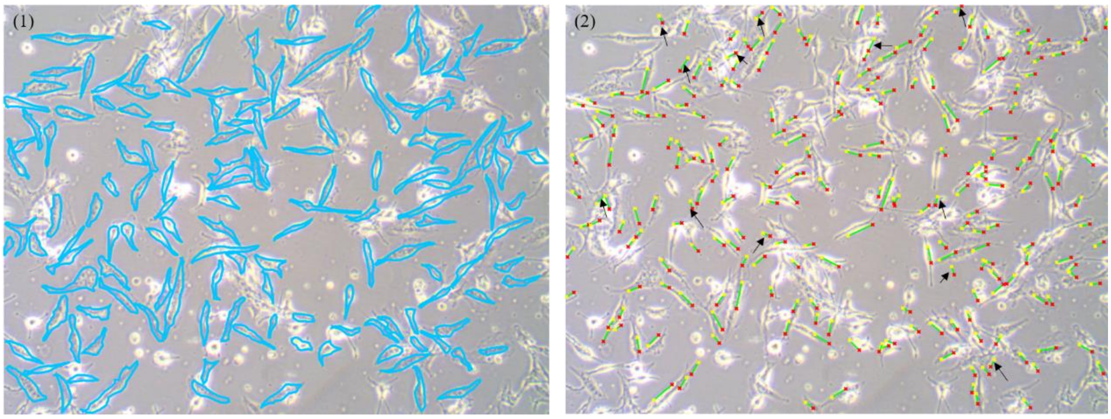

Figure S1. The visual interpretation of cells and algorithm detected cells in five cases. **a**(1)~**e**(1): the visual interpretation of cells in 1% CSE-Day 2, 2% CSE-Day 2, LPS 100 ng/mL-Day 2, LPS 500 ng/mL -Day 2 and TGF-β-Day 2 groups, respectively. **a**(2)~**e**(2): the algorithm detected cells in 1% CSE-Day 2, 2% CSE-Day 2, LPS 100 ng/mL-Day 2, LPS 500 ng/mL -Day 2 and TGF-β-Day 2 groups, respectively. The black arrows were represented wrong detections. (***Notice***: the different image color among five groups was due to different shooting lights and it had little impact through image processing).

Specifically, the number of different detections in five groups and the percentage were demonstrated in Figure S2. More than sixty percent of valid cells were detected in each case

which made our detection meaningful in statistics (Light blue color). However, nearly thirty percent of cells were not detected (Gray color) and small part of wrong detections were not real cells (Orange color) or repeat mark.

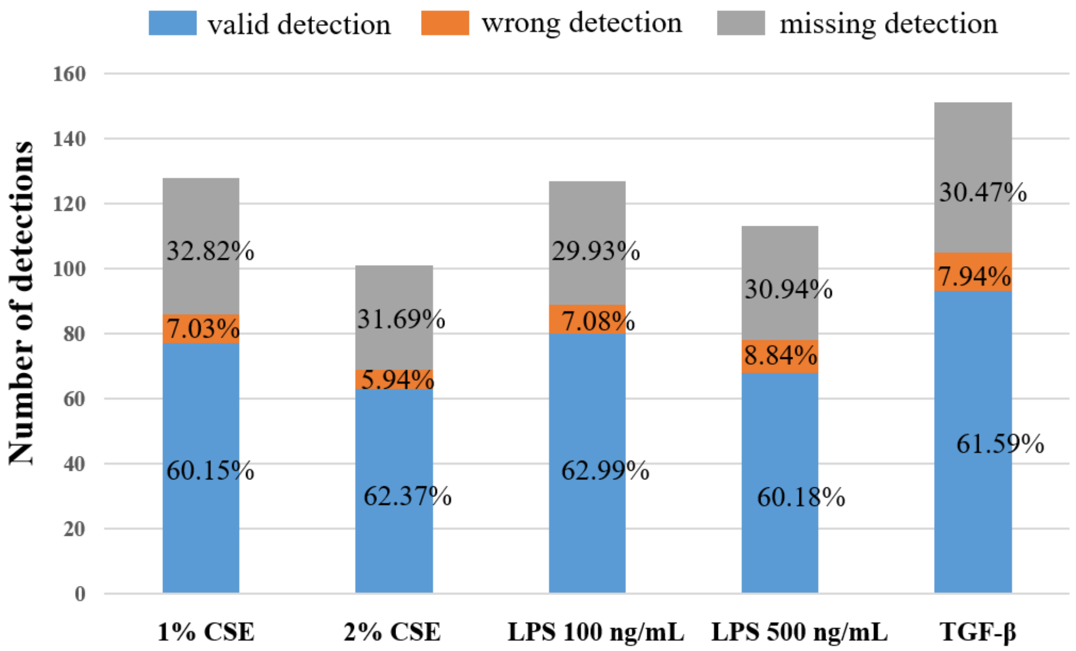

Figure S2. The number of detections including valid detections (Light blue color), wrong detections (Orange color) and missing detections (Gray color) in five groups. The percentage of each part was marked on the bar diagram.

Moreover, we measured the length of cells. It was important to note that the maximum distance between two points of cell boundary was defined as the real length of cells. As mentioned before, we printed the images in paper for measuring easily, thus the unit of length was millimeter (images were enlarged based on the same magnification). We displayed the real length of cells and the corresponding detected length in following scatter plot (Figure S3). The different color in the base map indicated the detection errors (error %= (real-detected)/real*100) and it showed that the detected lines seemed to be slightly underestimated in all five cases. The reason was that some cell antennas were difficult to detect by the proposed method as shown in Figure S1. Fortunately, most of the points ('+') distributed in low error area. We also calculated the average error of five groups as well as standard deviation (Figure S4). The average errors in five group were 9.59% (1% CSE), 9.71% (2% CSE), 10.21% (LPS 100 ng/mL), 8.61% (LPS 500 ng/mL) and 10.34% (TGF-β), respectively. Moreover, there was no significant difference in statistics analysis. Therefore, we

proposed that our method was effective in these cases.

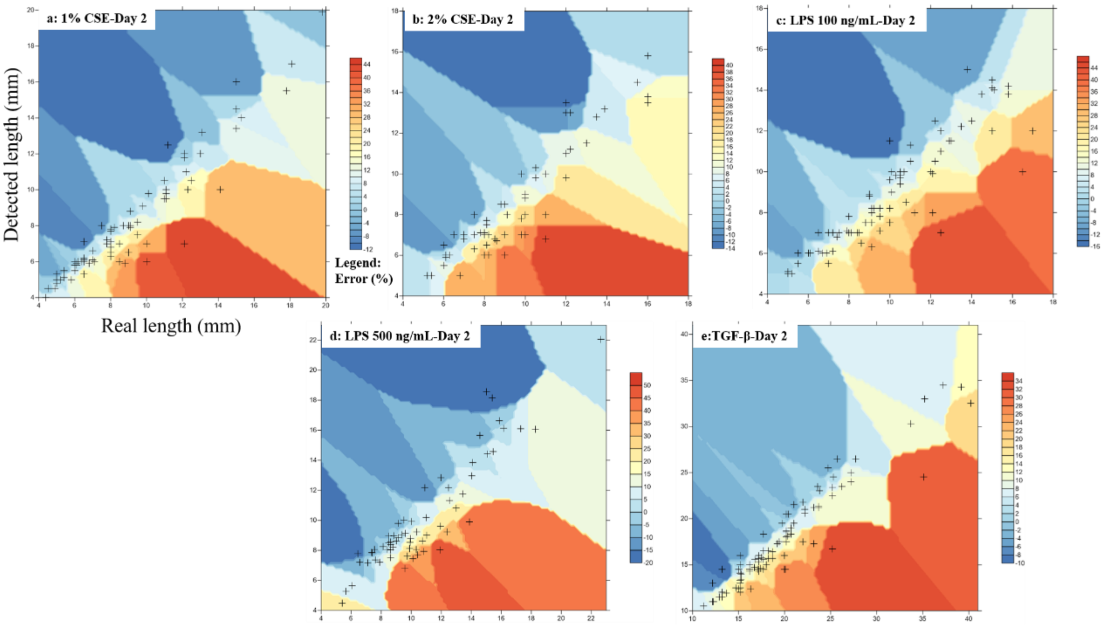

Figure S3. The scatter plots of real length and detected length in five groups. According to the Legend Error (%), the points were appeared in colorful base map to get the detail value of errors.

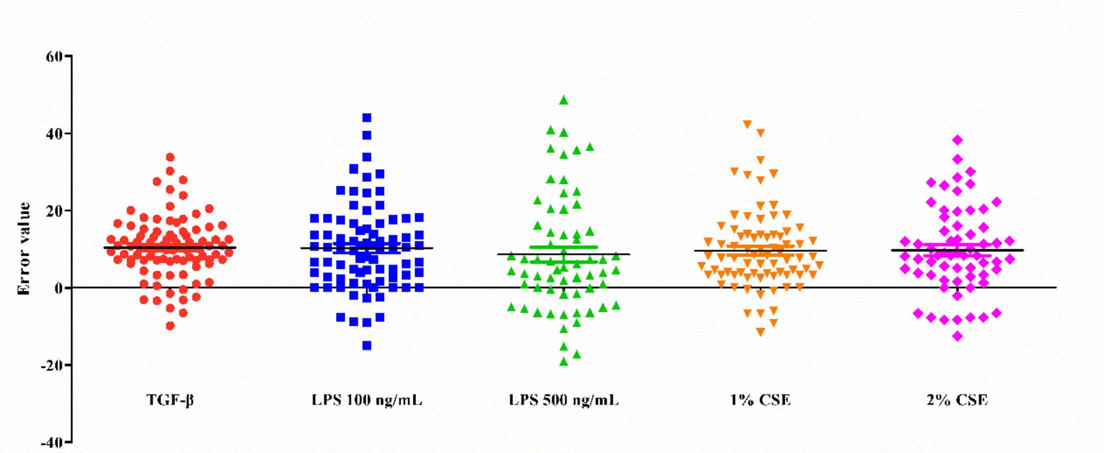

Figure S4. A scatter dot plot of length error comparing the five different groups (TGF-β; LPS 100 ng/mL and 500 ng/mL; 1% and 2% CSE groups) at day 2. Statistical differences between the different groups were evaluated using one-way ANOVA with Bonferroni's multiple comparison with GraphPrism 5 software.

Part 2: Confirmation of Arch Structure

According to the Figure 4a,c, we observed the shape is arch structure in TGF-β group. To confirm the shape, we performed the statistical analysis after the administration of TGF-β, LPS (100 ng/ml; 500 ng/ml) and CSE (1% ; 2%) for every 24 hours, we chose three points as it showed in Figure S5 of two independent experiments. Each experiment had performed in triplicate. It showed that the variations in the curve were significantly different in Figure S6. But in this case,

there was no significantly different between of TGF-β group at day 2 and day 3. So we further detect the slope between two points. In fact, we used slope to process the cell index curve which was showed in the Figure 4b&d by the RTCA system software. We also compared another two independent experiments results about the slope between different time intervals showed in Figure S7. We found the significantly difference between 0-24 h and 24-48 h after administration of TGF-β group and 2% CSE group (# represented the 24-48 h groups vs. 0-24 h groups). Additionally, the TGF-β group, 1% and 2% CSE groups showed significantly changes with control group in both 0-24 h and 24-48 h time intervals (* represented the groups vs. control group in different time intervals). Finally, we observed the statistically difference between the others group vs. TGF-βgroup at both 0-24 h and 24-48 h after administration except LPS 500 ng/mL group at 24-48h after administration (& represented LPS (100 ng/ml; 500 ng/ml) and CSE (1%; 2%) groups vs. TGF-β group in different time intervals).

In Figure 6, we further compared the different groups vs. control group, and shown the difference between TGF-β group and other groups in the modified Figure 6, all analysis confirmed that the arch structure is specific character for TGF-β on epithelia cell.

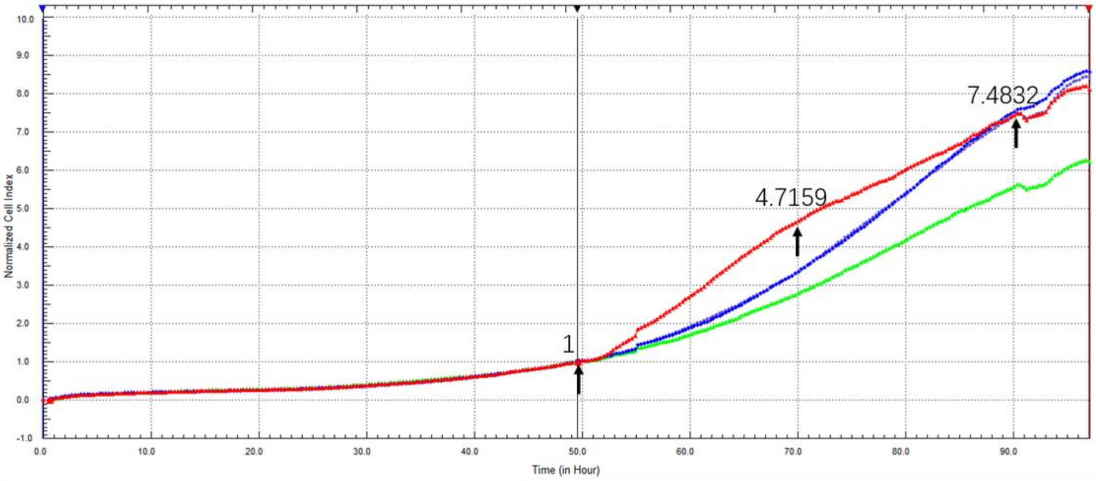

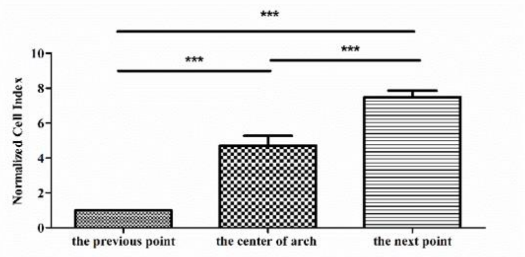

	Cell index ± SD
Previous point	1
The center of arch structure	4.7159±0.5437
Next point	7.4832±0.3751

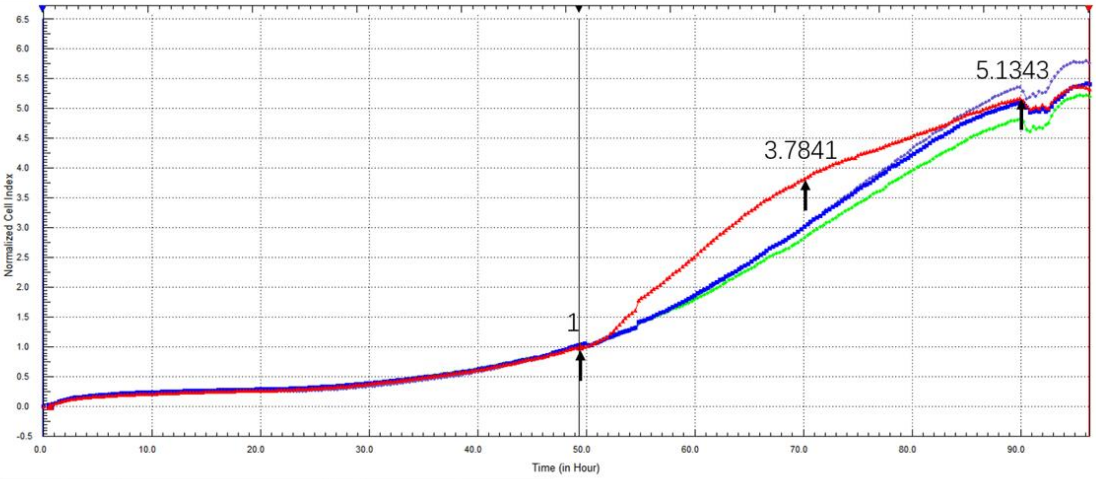

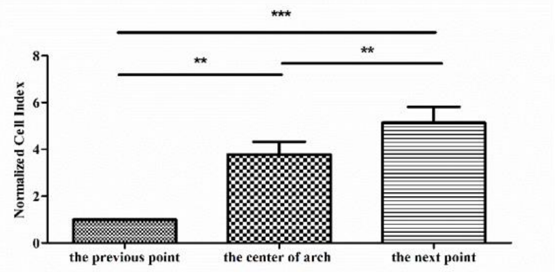

	Cell index ± SD
Previous point	1
The center of arch structure	3.7841±0.5438
Next point	5.1343±0.6798

Figure S5: The specific value of three points (the center of arch structure, the previous and next points) in two independent experiments (N=3). The table showed the specific value of cell index ± SD. Statistical differences between the different groups were evaluated using one-way ANOVA with Bonferroni's multiple comparison with GraphPrism 5 software. $p<0.05$ was considered significant. *** $p<0.001$, ** $p<0.01$.

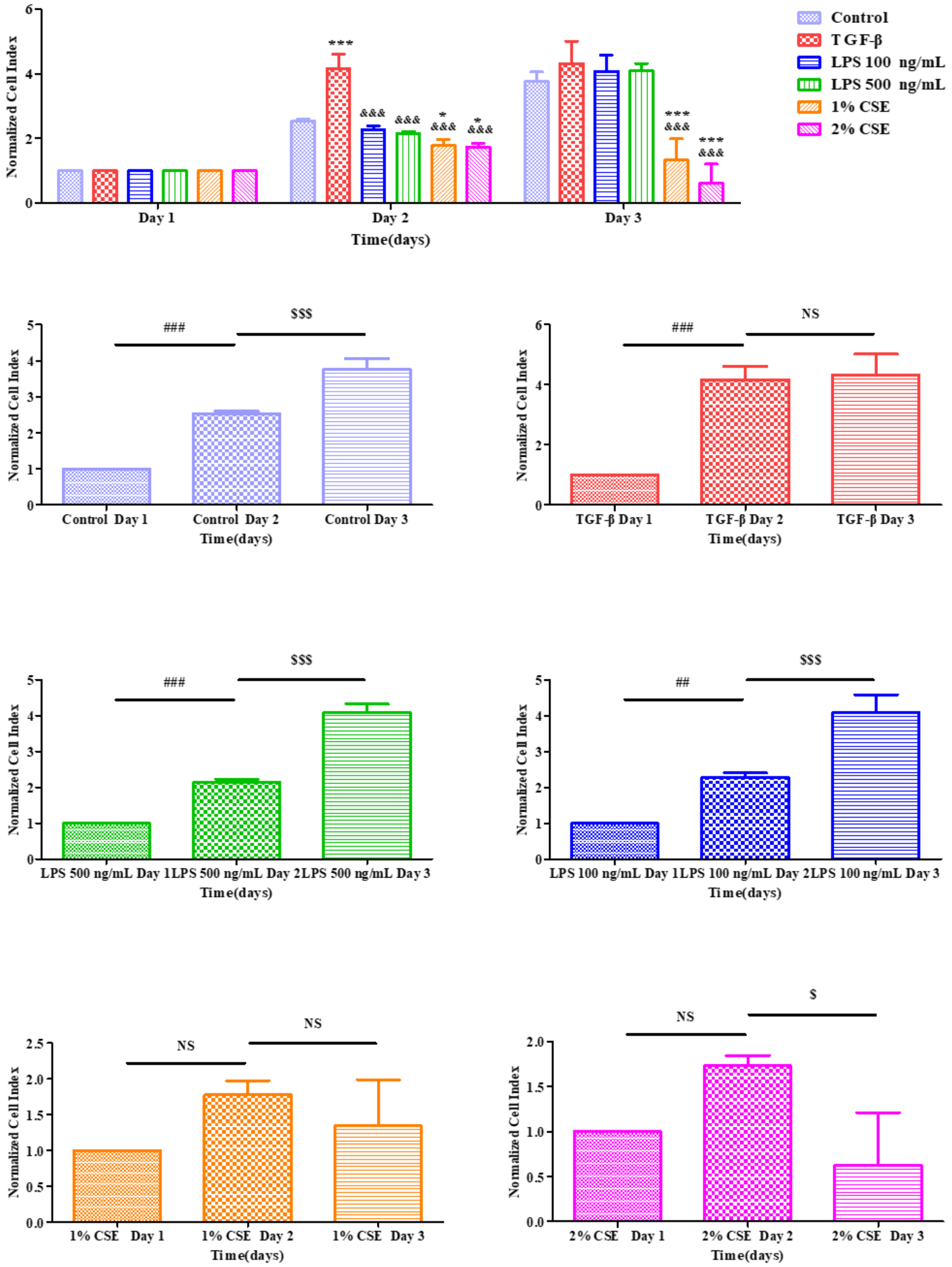

Figure S6: The specific value of normalized cell index at Day 1 to Day 3. (N=3). Statistical differences between the different groups were evaluated using one-way ANOVA with Bonferroni's multiple comparison with GraphPrism 5 software. $p<0.05$ was considered significant. *** $p<0.001$, * $p<0.05$ vs control group in different time points. # represented groups at Day 1 vs. groups at Day 2. ### $p<0.001$, ## $p<0.01$. $ represented groups at Day 2 vs. groups at Day 3. $$$ $p<0.001$, $ $p<0.05$. &&& $p<0.001$ vs. TGF-β group in different time points.

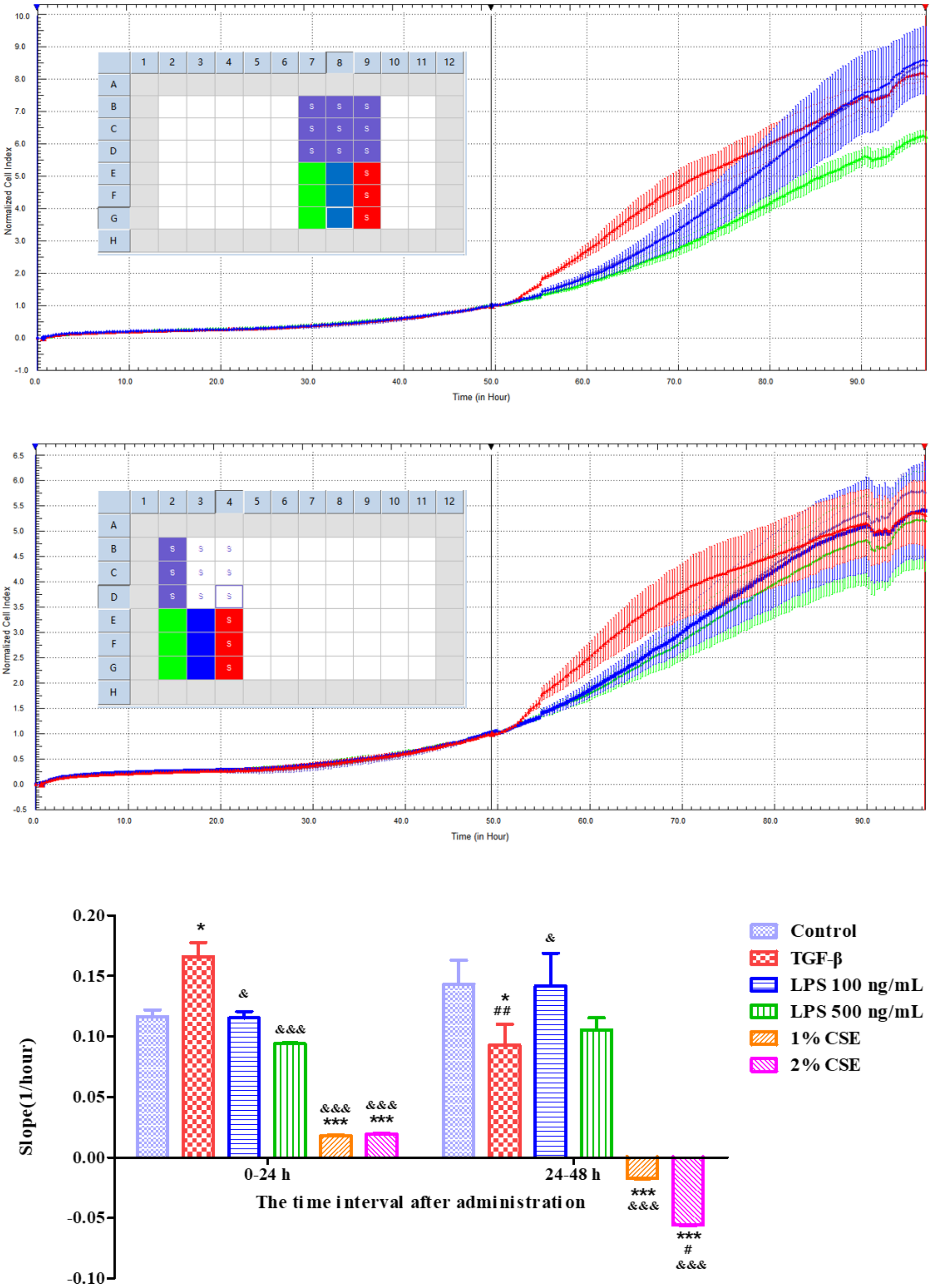

Figure S7: The slope at 0-24h and 24-48h after administration. (N=6). Statistical differences between the different groups were evaluated using one-way ANOVA with Bonferroni's multiple comparison with GraphPrism 5 software. p<0.05 was considered significant. * p<0.05 vs control group in different time intervals. # represented the 24-48h groups vs. 0-24h groups. ## p<0.01, # p<0.05. &&& p<0.001, & p<0.05 vs. TGF-β group in different time intervals.

Part 3: Code

Pseudo-code for quantification of cells' boundary based on MATLAB R2014a

I: Image preprocessing

```
load ('Original_image.tif'); % Input original three-channel image (RGB);
Gray_image=rgb2gray(Original_image);% Transform to gray image;
Enhanced_image=((Gray_image/255)^3)*255; % Contrast manipulation based on cubic function;
Denoised_image=medfilt2(Enhanced_image, [3,3]); % Denoise by median filter, window size: 3*3;
% Canny edge detection
Threshold =[0.02, 0.2];
Sigma=4;
Edge_image=edge(Denoised_image, 'canny', Threshold, Sigma);
```

II: Hough transform

% The minimal interval of ρ and θ was defined as 0.5; The minimal detected lines were set as 20 pixels.

[H_{ij}, ρ_i, θ_j]=hough(Edge_image, 'RhoResolution', 0.5, 'Theta',0: 0.5: 179.5);

Peaks=houghpeaks(H_{ij}, 1000,'threshold', ceil(0.3*max(H_{ij}(:)))); % Find the peaks in matrix H_{ij} which contains the row and column coordinates of the HT bins.

Detected_lines=houghlines(Edge_image, ρ_i, θ_j, Peaks, 'MinLength', 20); % Extract line segments in the Edge_image associated with particular bins in HT.

III: Post processing

```
% Integrate the lines with intersections as a longer polyline.
 for i=1:number of Detected_lines
     for j=i: number of Detected_lines
         if the minimal length between Detected_lines (i) and Detected_lines (j) <5 pixels
             Integrate them as one polyline:
             polylines=norm(Detected_lines (i))+ norm(Detected_lines (j));
         end
     end
 end

% Remove excessive lines to guarantee a cell represented by one line segment
 for i=1:number of Detected_lines
     for j=i: number of Detected_lines
         if the minimal length between Detected_lines (i) and Detected_lines (j) <15 pixles &&
            the included angle |θi -θj |<20°
             Record the maximal one:
             max_Lines=max(norm (Detected_lines (i)), norm (Detected_lines (j)));
         end
     end
 end
 The final detected lines are the combinations of polylines and max_Lines.
```

Output: numbers of final detected lines and lengths of detected lines.
